# Supplementary figures and images for: SARS-CoV-2-Induced TSLP Is Associated with Duration of Hospital Stay in COVID-19 Patients
Source: Viruses. 2023 Feb 17;15(2):556. doi: 10.3390/v15020556 (PMC9959394; doi:10.3390/v15020556)

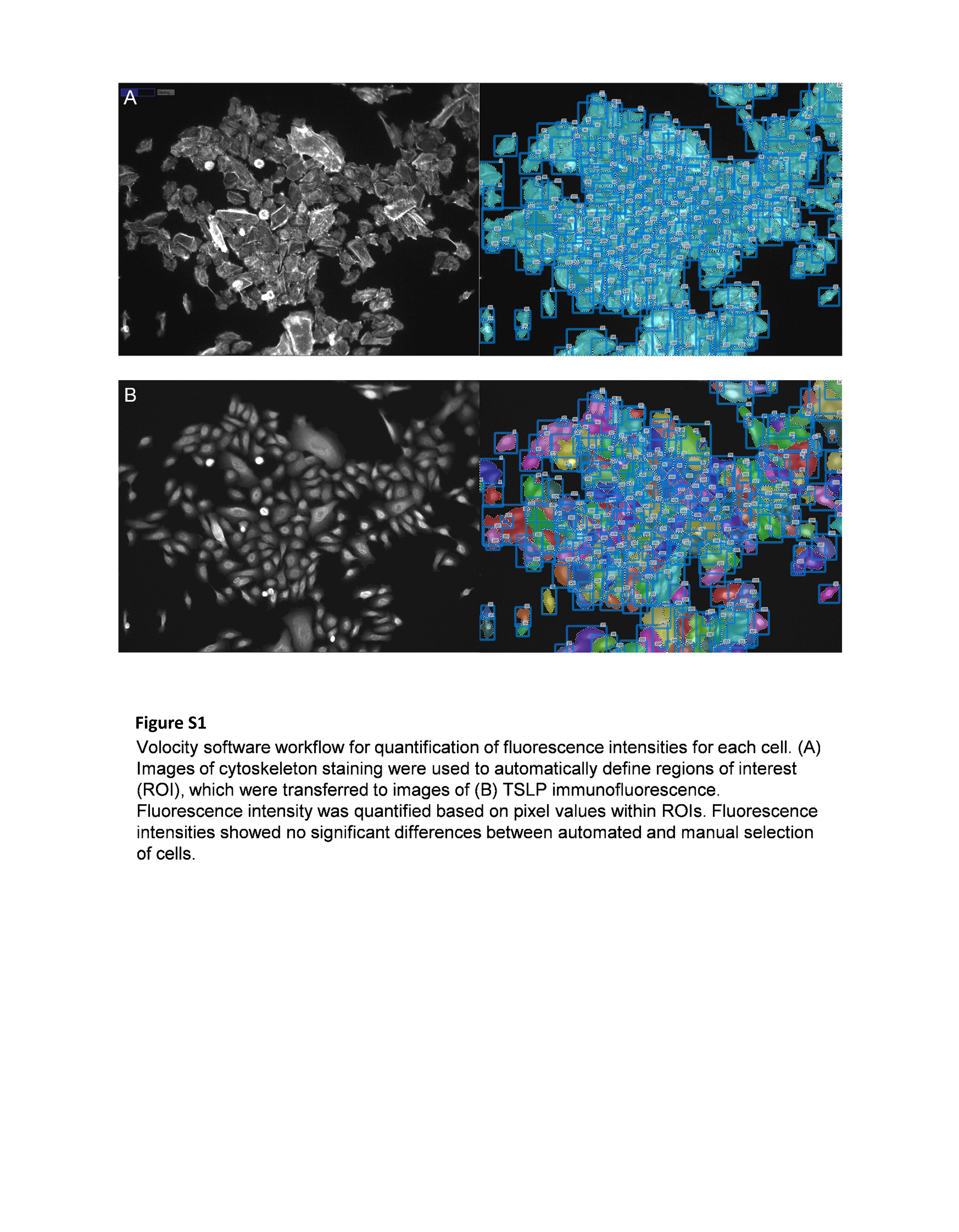

Supplement: Supplementary file 1 [file viruses-15-00556-s001.zip › Camera Roll/S1.png]

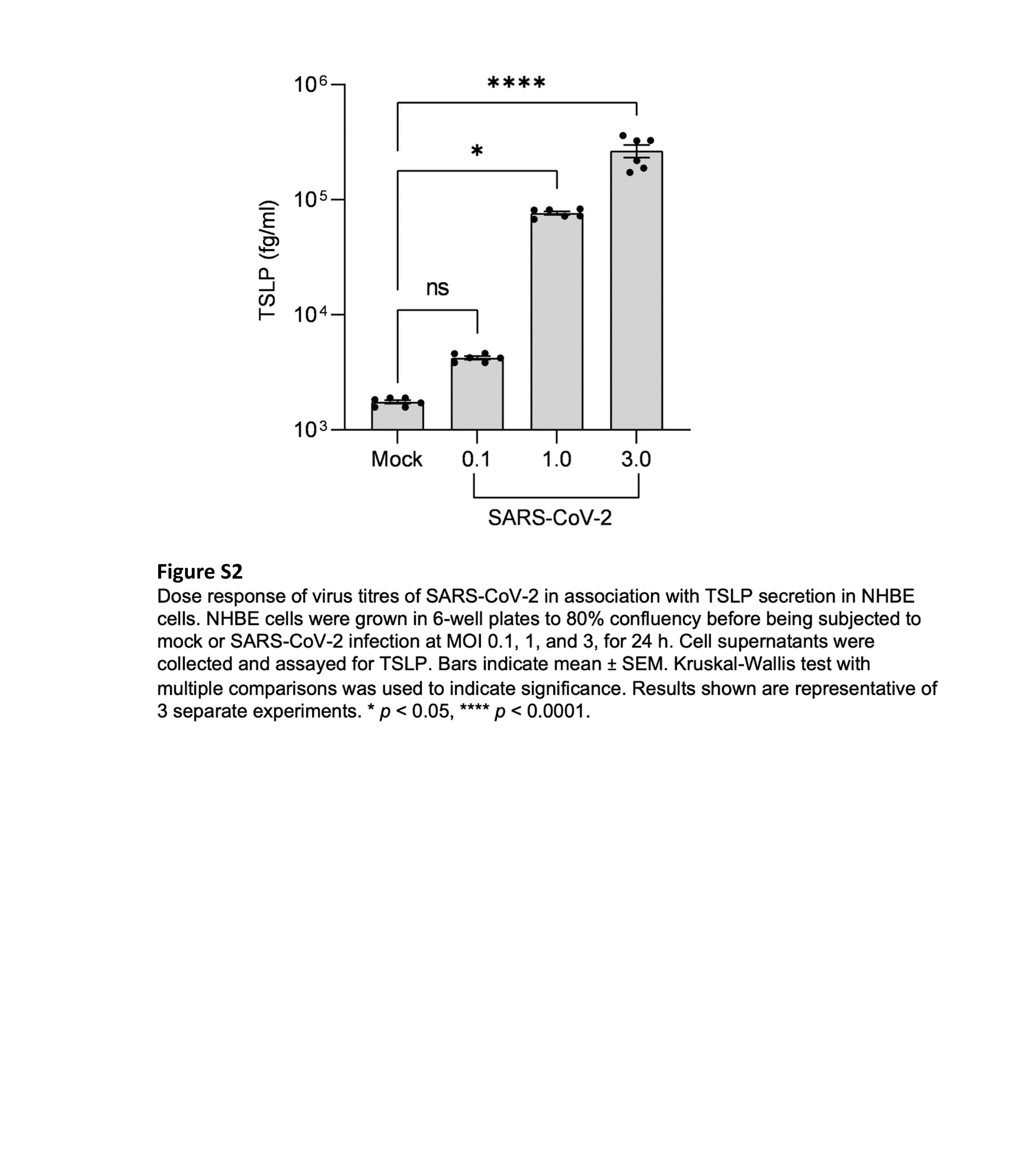

Supplement: Supplementary file 1 [file viruses-15-00556-s001.zip › Camera Roll/S2.png]

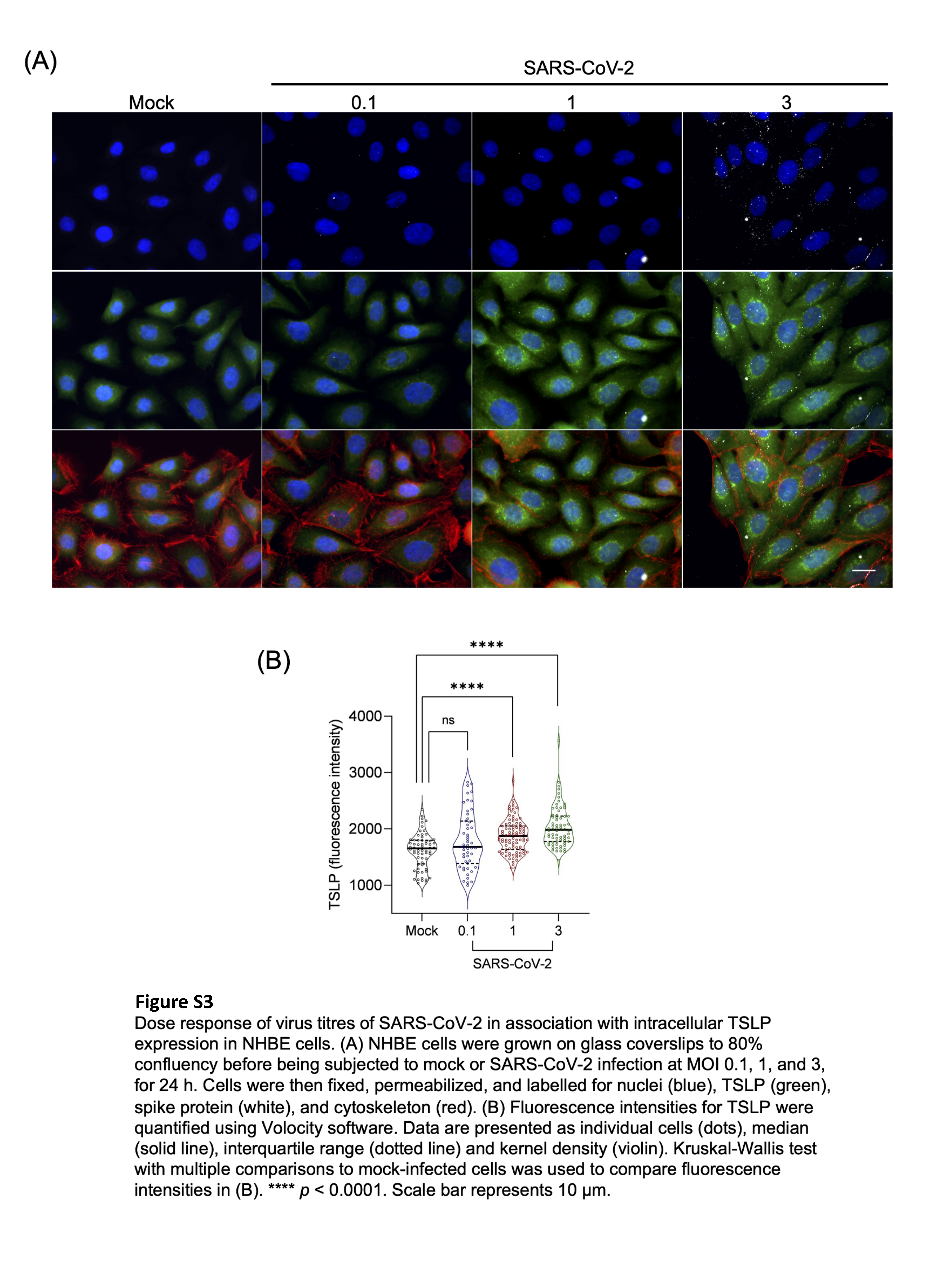

Supplement: Supplementary file 1 [file viruses-15-00556-s001.zip › Camera Roll/S3.png]

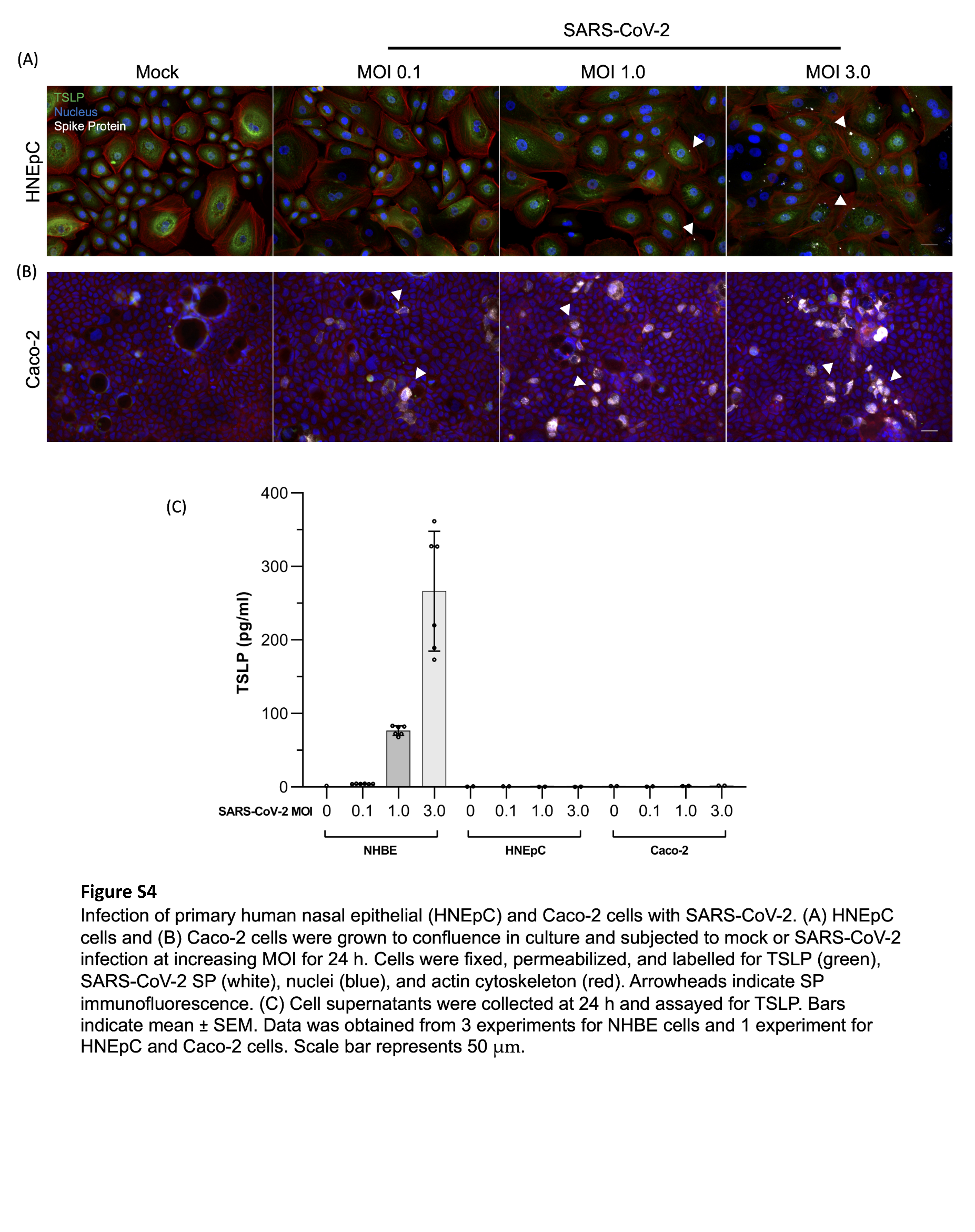

Supplement: Supplementary file 1 [file viruses-15-00556-s001.zip › Camera Roll/S4.png]
